# Supplementary material for: Market framing bias and cross-sectional stock returns
Source: PLoS One. 2023 Aug 30;18(8):e0290500. doi: 10.1371/journal.pone.0290500 (PMC10468084; doi:10.1371/journal.pone.0290500)
Supplement: Attachment — Submitted filename: Responses to the reviewers.docx [file pone.0290500.s002.docx]

Responses to the reviewer

PLOS ONE

We respond to the comments, shown in red, below. We use the letter R to indicate our response.

Journal Requirements:

When submitting your revision, we need you to address these additional requirements.

1. Please ensure that your manuscript meets PLOS ONE's style requirements, including those for file naming. The PLOS ONE style templates can be found at
   https://journals.plos.org/plosone/s/file?id=wjVg/PLOSOne_formatting_sample_main_body.pdf and 
   <https://journals.plos.org/plosone/s/file?id=ba62/PLOSOne_formatting_sample_title_authors_affiliations.pdf>

**R**: We re-formatted the paper according to the PLOS ONE style template to meet the requirements of PLOS ONE.

2. We suggest you thoroughly copyedit your manuscript for language usage, spelling, and grammar. If you do not know anyone who can help you do this, you may wish to consider employing a professional scientific editing service.

Upon resubmission, please provide the following: 
● The name of the colleague or the details of the professional service that edited your manuscript
● A copy of your manuscript showing your changes by either highlighting them or using track changes (uploaded as a *supporting information* file)
● A clean copy of the edited manuscript (uploaded as the new *manuscript* file)

**R:** We asked a professor (HZ. Zeng) who had published papers in *Management Science* to help us with copyedit. And we hired Editsprings to make numerous revisions to the paper, including language usage, spelling, and grammar. Details can be queried from the supporting information file.

We have also uploaded the supporting information file and the new manuscript file as requested.

3. In your Data Availability statement, you have not specified where the minimal data set underlying the results described in your manuscript can be found. PLOS defines a study's minimal data set as the underlying data used to reach the conclusions drawn in the manuscript and any additional data required to replicate the reported study findings in their entirety. All PLOS journals require that the minimal data set be made fully available. For more information about our data policy, please see <http://journals.plos.org/plosone/s/data-availability>.

"Upon re-submitting your revised manuscript, please upload your study’s minimal underlying data set as either Supporting Information files or to a stable, public repository and include the relevant URLs, DOIs, or accession numbers within your revised cover letter. For a list of acceptable repositories, please see http://journals.plos.org/plosone/s/data-availability#loc-recommended-repositories. Any potentially identifying patient information must be fully anonymized.

**R:** Upon re-submitting our revised manuscript, we upload our study’s minimal underlying data set as Supporting Information files, please check it.

4. Please ensure that you include a title page within your main document. You should list all authors and all affiliations as per our author instructions and clearly indicate the corresponding author.

**R:** Upon re-submitting our revised manuscript, we upload a title page within the document, which lists all authors and all affiliations as per our author instructions and we clearly indicate the corresponding author.

Additional Editor Comments:
I thank the authors for submitting their work to PLOS ONE.
Before I would send the paper to reviewers, to give the paper a fair chance, I would like the authors to address the following issues:
1. theoretical rationale. It is not clear to me how exactly we can use the up vs down beta as a measure of the framing bias among investors. This needs to be explained in more detail. the authors say "Glascock and Lu-Andrews (2018) discover that the beta coefficients of the market are different under different market states, which is probably due to the framing effect.
This evidence reveals that the framing effect in a stock market can be represented by the
difference between beta in different market statuses (up or down markets), which inspires us to use the absolute difference between betas in the up and down markets to measure the framing effect." The key, and worrying, term is PROBABLY here. As a rationale it is too weak. the reader needs to be reasonably strongly convinced that the measure used is most likely measuring what it is suppose to measure.

**R:** We give three reasons in Section 2 (Quantification of MFB) to describe why we can use the absolute difference between betas in the up and down markets to measure the market framing effect. And in the revised version, we improved these three reasons to make them better express our views. The revised version also improved the description of “PROBABLY”.

We put some of the improved excerpts below:

First, the up or down market provides a “natural frame” to characterize the investment climate. Kahneman (2003) points out that the framing effect is the anomaly that extensionally equivalent descriptions lead to different choices by altering the relative salience of different aspects of the problem (different environments). For investors, as observed by Glaser et al. (2007), a positive frame (profit-making environment) or a negative frame (loss-making environment) may naturally indicate the up or down market. In other words, our definition of such a bias as MFB is similar to Kahneman's (2003) definition of framing bias, except that our bias is produced under a different market framework. That's why we call it MFB, instead of just calling it framing bias.

Second, we use the difference between the betas in the up and down markets to measure the behavioral bias of investors. Glascock and Lu-Andrews (2018) found that the market beta coefficients changed under different market states, which could be attributed to the framing effect. This evidence reveals that the framing effect in a stock market can be reflected by the difference between beta in different market situations (up or down markets), which inspires us to use the absolute difference between betas in the up and down markets to measure the framing effect.

Finally, we take the absolute value, because the framing effect, as defined by Kahneman (2003), has no positive or negative direction. It occurs when different descriptions of the framework lead to irrational decisions of investors. According to CAPM theory (Sharpe, 1964; Lintner, 1965), rational investors should have the same beta in both up and down markets. If the beta values differ across market status (frameworks), it reflects the existence of a framing effect for investors.”

2. one would expect an empirical investigation into if the proposed factor is a priced risk factor in equilibrium, this does not seem to have been done? Ie accounting for the well known factors (eg FF5) is the proposed effect still attracting a significant risk premium?

R: To the best of our knowledge, previous studies on framing effect focused on discussing the existence of framing effect. We are the first to measure the “market framing bias” for individual stocks, that seeks to use MFB as an individual stock characteristic and explore whether it can generate anomalies. Building a pricing factor based on MFB will be the topic of our next paper, because it is only possible to further study whether it is a common pricing factor after discussing the anomaly of MFB clearly. If we put the two topics in one paper, the paper would be too long, and the theme would not be clear enough. Therefore, in this paper, we mainly discuss whether “FMB” as an individual stock characteristic will generate excess returns that cannot be explained by pricing factors such as FF5, Q4, SL2 factors. The excess return reported in Table 3 and Table 4 of the paper is the average of the excess returns of the corresponding portfolios, that is, the risk premium of the corresponding portfolios (see Bodie et al. 2022, Essentials of Investment, McGraw Hill LLC). The alpha then calculated is the risk premium adjusted for factors such as FF5. The source of the method in this paper can refer to Empirical Asset Pricing (Bali, et al. 2016, Published by John Wiley & Sons, Inc., Hoboken, New Jersey).

3. there is a substantial literature on betas in up vs down markets, one would expect that some(alternative) explanations have been proposed for the betas difference. how does this paper differentiates between different explanations for differences in up vs down betas?

R: There is a large amount of literature that studies the beta coefficients in rising and falling markets, but they mainly explain the difference in beta coefficients by the different risk preferences of investors in rising and falling markets. This paper is different from them, and defines the difference in investors’ return predictions (beta) under different scenarios (frames) in rising and falling markets as MFB. The framing effect is an anomaly that extensionally equivalent descriptions lead to different choices by altering the relative salience of different aspects of the problem (Kahneman, 2003). FB contains many aspects, and this paper only analyzes it from the different frames of market rising and falling, so it is defined as MFB. Before this, the frame effect was mainly measured by questionnaire surveys, and this paper gives an objective method to measure it using public trading data, which provides important reference significance for the quantitative research of behavioral finance, and has great innovation.
