# Supplementary material for: Market framing bias and cross-sectional stock returns
Source: PLoS One. 2023 Aug 30;18(8):e0290500. doi: 10.1371/journal.pone.0290500 (PMC10468084; doi:10.1371/journal.pone.0290500)
Supplement: Attachment — Submitted filename: Reviewer comments.docx [file pone.0290500.s003.docx]

**Reviewer comments**

**Rationale for MFB as a Measure of Framing Effect:** The manuscript asserts that MFB is a measure of the framing effect observed in the stock market. However, the justification for this claim is not thoroughly discussed. The authors briefly mention that the framing effect in behavioural finance is a cognitive bias based on whether investors are in a profit-making or loss-making environment. ***It would be beneficial to provide a more comprehensive explanation of how MFB captures this framing effect and the underlying mechanisms through which it influences investor decision-making***

**Data**

The sample period of January 2000 to December 2019 appears to be extensive, allowing for a comprehensive analysis of the research question. The inclusion criterion of stocks traded for at least 36 months during the sample period is reasonable and helps ensure a sufficient number of observations. With 3804 stocks and nearly 500,000 firm-month observations, the sample size is substantial and provides a solid foundation for statistical analysis. Controlling for firm-specific characteristics is crucial in studying cross-sectional returns, and the manuscript lists several variables used for this purpose. The inclusion of beta, size, book-to-market equity ratio, short-term reversal, momentum return, illiquidity, co-skewness, downside beta, VOLDU, and VaR provides a comprehensive set of factors that may affect expected stock returns.

1. **Definition of MFB:** The manuscript introduces MFB as the bias of the risk-return trade-off between up and down markets. While the rationale for this definition is provided, it would be helpful to have a more explicit connection between MFB and the framing effect. *The authors briefly mention that MFB reflects how investor expectations differ in the gain/loss framework, but a deeper discussion on how MFB captures framing effects and cognitive biases would enhance the understanding of the concept.*
2. **Model Specification**: Equation (1) represents the model used to estimate the difference between betas in up and down markets. However, the authors should provide a more detailed explanation of the variables and parameters in the equation. *Specifically, they should clarify the definitions of R, f, M, and ε, and explain how these variables are calculated or sourced. Providing more clarity on the model specification would improve the reproducibility and transparency of the methodology.*
3. **Justification for Absolute Difference**: The authors justify the use of the absolute difference between betas by stating that the framing effect has no positive or negative direction. While this reasoning is understandable, it would be valuable to discuss potential drawbacks or limitations of this approach. *Are there any scenarios or implications where considering the direction of the difference could provide additional insights? Discussing alternative approaches and their respective advantages and disadvantages would strengthen the methodology.*
4. **Empirical Evidence and Validation**: The manuscript states that MFB may convey information useful for predicting future stock returns. However, no empirical evidence or validation of this claim is provided. It is important to include results from statistical tests or regression analyses that demonstrate the predictive power of MFB in forecasting future returns. Additionally, discussing the robustness of the findings and potential confounding factors would enhance the credibility of the methodology.
5. Comparison with Previous Studies: The authors briefly mention Levi and Welch (2020) as a previous study, but more discussion and comparison with relevant literature is needed. How does the proposed methodology differ from and build upon existing approaches to measuring framing effects and behavioural biases in financial markets? Highlighting the unique contributions and advantages of the current methodology would strengthen the manuscript's originality.

In summary, while the manuscript presents a methodology for quantifying MFB, there are several areas that require further clarification, empirical evidence, and comparative analysis. Addressing these points would improve the overall strength and reliability of the methodology section.

1. **Methodological Details:** The manuscript lacks detailed explanations of the specific calculations and data sources used to measure MFB and construct the decile portfolios. Providing step-by-step explanations of the calculations, including the formulas used and the specific databases or sources of data, would enhance the replicability of the study.
2. **Interpretation of Results**: While the summary table provides the excess returns and abnormal returns for each decile portfolio, the manuscript lacks a comprehensive interpretation and discussion of the findings. It is essential to provide a deeper analysis of the results, including their statistical significance, economic significance, and potential implications for investors. Additionally, connecting the findings back to the literature on the framing effect and behavioral finance would strengthen the manuscript's contribution.
3. **Bivariate Analysis:** The manuscript briefly mentions a bivariate portfolio analysis but does not provide detailed results or discussion of this analysis. It would be valuable to present the findings of the bivariate analysis and explore the relationship between MFB and other firm-specific characteristics or behavior-related asset pricing factors. This would provide a more comprehensive understanding of the factors that influence MFB and their impact on stock returns.
4. **Limitations and Robustness:** The manuscript does not address potential limitations of the methodology or conduct robustness tests to assess the stability and reliability of the findings. *It is crucial to acknowledge any limitations of the study, such as data limitations or assumptions made, and discuss how these limitations may affect the interpretation of the results. Additionally, conducting sensitivity analyses or alternative model specifications would enhance the robustness of the findings.*
